# Efficacy and safety of canakinumab, a long acting fully human anti-Interleukin-1β antibody, in systemic juvenile idiopathic arthritis with active systemic features: results from a phase III study

**DOI:** 10.1186/1546-0096-9-S1-O21

**Published:** 2011-09-14

**Authors:** N Ruperto, H Brunner, G Horneff, P Quartier, T Constantin, Y Berkun, M Erguven, T Kallinich, R Brik, NM Wulffraat, MA Ferrandiz, L Rutkowska-Sak, H Ozdogan, L McCann, K Lheritier, R Preiss, L Tseng, A Martini, DJ Lovell

**Affiliations:** 1 PRINTO-IRCCS, Genova, Italy; 2 PRCSG, Cincinnati, USA; 3 Novartis Pharma AG, Basel, Switzerland; 4 Novartis Pharmaceuticals Corporation, NJ, USA

## Objectives

To assess the efficacy and safety of canakinumab, in systemic juvenile idiopathic arthritis (sJIA) with active systemic features at enrollment.

## Methods

In this 4-week randomized, controlled, double-blind study, sJIA patients received a single subcutaneous dose of canakinumab 4 mg/kg (maximum 300 mg) or placebo at Day 1. The primary objective was to show superior efficacy of canakinumab vs placebo in achieving an adapted ACR Ped30 (ACR criteria plus absence of fever) treatment response at Day 15.

## Results

In total, 84 patients (age 2–19 yrs) received treatment (canakinumab, n=43; placebo, n=41). Baseline demographics and characteristics were comparable between groups, except for age. The overall group means were: disease duration 3.4 yrs; CRP 200.6 mg/L (normal range 0-10 mg/L); number of active joints 14.1; and prednisone equivalent therapy 0.6 mg/kg/day. At Day 15, canakinumab was superior to placebo for the primary and secondary endpoints: ACR Ped30, 83.7 vs 9.8%; ACR Ped50, 67.4 vs 4.9%; ACR Ped100, 32.6% vs 0, respectively (all p<0.0001). ACR Ped30/50 responses with canakinumab remained significantly higher than with placebo at Day 29 (both p<0.0001). Six patients on canakinumab and 37 patients on placebo discontinued due to unsatisfactory therapeutic effects. Adverse events (AEs) occurred in 55.8% of canakinumab and 39.0% of placebo-treated patients. No discontinuations occurred due to AEs. Two non-fatal serious AEs were reported in each group.

## Conclusions

Canakinumab has superior efficacy to placebo in sJIA, providing rapid onset of action and robust response (at least ACR Ped50 with fever disappearance) in the majority of patients.

## Disclosure of Interest

**K. Lheritier** Shareholder of: Novartis, Employee of: Novartis; **R. Preiss** Shareholder of: Novartis, Employee of: Novartis; **L. Tseng** Shareholder of: Novartis, Employee of: Novartis

